# Positive schizotypy is associated with amplified mnemonic discrimination and attenuated generalization

**DOI:** 10.1192/j.eurpsy.2022.409

**Published:** 2022-09-01

**Authors:** Á. Vass, M. Becske, Á. Szőllősi, M. Racsmány, B. Polner

**Affiliations:** 1 Semmelweis University, Department Of Psychiatry And Psychotherapy, Budapest, Hungary; 2 Budapest University of Technology and Economics, Department Of Cognitive Science, Budapest, Hungary; 3 Research Centre for Natural Sciences, Institute Of Cognitive Neuroscience And Psychology, Budapest, Hungary

**Keywords:** episodic memory, schizotypy, pattern separation, pattern completion

## Abstract

**Introduction:**

Tendency to experience inaccurate beliefs alongside perceptual anomalies constitutes positive schizotypal traits in the general population and shows continuity with the positive symptoms of schizophrenia. It has been hypothesized that the positive symptomatology of schizophrenia, and by extension, positive schizotypy, are associated with specific alterations in memory functions. Imbalance between memory generalization and episodic memory specificity has been proposed on several counts; however, the direction of the imbalance is currently unclear.

**Objectives:**

We aimed to contrast two competing hypotheses regarding the association between positive schizotypy, and memory alterations in a general population sample (N=71) enriched for positive schizotypy from a larger pool of individuals (N=614).

**Methods:**

Positive schizotypy was measured with the short-version of the O-LIFE questionnaire, and memory specificity and generalization was captured by the well-established Mnemonic Similarity Task.

**Results:**

Distortions in the behavioural memory performance indices were found to correlate with positive schizotypy: individuals prone to unusual experiences demonstrated increased discrimination and reduced generalization (explaining 10% and 17% of variance, respectively). Associations were robust when controlled for the disorganized, negative and impulsive-asocial dimensions of schizotypy and associated psychopathology.

**Conclusions:**

Our findings show that people who are prone to irrational beliefs and unusual experiences also show measurable alterations in memory and likely have difficulty grasping the global picture and rather be overpowered by fragments of information.

**Disclosure:**

No significant relationships.

